# Effects of dietary supplementation with purple garlic powder and oregano essential oil on intestinal health in post-weaning piglets from commercial farms

**DOI:** 10.1007/s11259-022-10053-2

**Published:** 2022-12-15

**Authors:** Daniel Serrano-Jara, Jorge Rivera-Gomis, José Antonio Tornel, Antonio Bernabé, Cristina Martínez-Conesa, José Antonio Navarro, Ricardo Cánovas, Julio Otal, María José Cubero

**Affiliations:** 1 Department of Comparative Anatomy and Pathology, Veterinary Medicine Faculty, Regional Campus of International Excellence “Campus Mare Nostrum”, University of Murcia, 30100 Murcia, Espinardo Spain; 2 Animal Health Department, Veterinary Medicine Faculty, Regional Campus of International Excellence “Campus Mare Nostrum”, University of Murcia, 30100 Murcia, Espinardo Spain; 3 Present Address: Centre for Epidemiology and Planetary Health, Department of Veterinary and Animal Science, Northern Faculty, Scotland’s Rural College, An Lòchran, 10 Inverness Campus, Inverness, IV2 5NA Scotland UK; 4 Dalland Hybrid España, S.A., Fortuna, Murcia Spain; 5 Research Group On Rainfed Agriculture for Rural Development, Department of Rural Development, Oenology and Sustainable Agriculture, Murcia Institute of Agri-Food Research and Development (IMIDA), 30150 Murcia, La Alberca de Las Torres Spain; 6 Animal Production Department, Regional Campus of International Excellence “Campus Mare Nostrum”, University of Murcia, 30100 Murcia, Espinardo Spain

**Keywords:** Piglets, Garlic, Oregano, Intestinal health, Growth Performance

## Abstract

This work studied the effects of the inclusion of Purple Garlic Powder (PGP) and Oregano Essential Oil (OEO) in the feed, at different doses and combinations, on intestinal health and the growth performance of 140 and 3000 piglets, respectively, weaned at 21 days of age. Seven dietary treatments were used: a negative control group (basal diet), a positive control group with ZnO (3000 mg/Kg of feed), two groups with OEO at 0.4% and 1.2% respectively, two groups with PGP at 0.4% and 2% respectively and one group with OEO at 1.2% combined with PGP at 2%. Only the positive control group received ZnO in the diet. Each group of piglets received the treatment for seven weeks, from weaning, and were later sacrificed to obtain jejunum and ileum samples for counting of goblet cells, intraepithelial lymphocytes, and IgA-producing cells. The growth performance were measured at the beginning and at the end of the seven weeks. In jejunum and ileum, the number of goblet cells increased in the groups with ZnO, PGP 2%, OEO 1.2% and PGP 2% + OEO 1.2%, presenting significant differences with the rest of the groups. The results obtained for the intraepithelial lymphocyte count were in line with those obtained for the count of goblet cells. Regarding IgA-producing cells, the groups that showed significantly favourable results in the jejunum and ileum were OEO 1.2%, PGP 2% and their combination, but the groups that showed the most similar means to ZnO were the OEO 0.4% and the PGP 0.4%. Regarding the growth performance, PGP 2%, OEO 1.2% and their combination had similar results to ZnO. The intestinal health of piglets could be improved, without harming the growth performance, by means of the supplementation of PGP 2%, OEO 1.2% and PGP 2% + OEO 1.2% offering a natural alternative to the use of ZnO.

## Introduction

The weaning period is a critical life stage in the pig production cycle. The immature immune system, the stress associated with separation from the mother, and changes in feeding and the environment adversely affect the endocrine functions, growth and animal welfare of weaned piglets (Moberg and Mench 2000). All these factors have a negative influence on the piglet and can lead to intestinal dysfunctions (Zhu et al. 2012) due to the proliferation of pathogenic bacteria (*Escherichia coli*) and inflammation of the mucous membranes, resulting in a decrease in digestive performance (Pié et al. 2004; Wijtten et al. 2011; Kim et al. 2012a); and, therefore, a reduction in the absorption of nutrients, significant growth delays and great economic losses.

The addition of zinc oxide (ZnO) at therapeutic doses (2000 to 4000 mg/Kg) in piglets’ diet during the post-weaning period has been widely practiced in commercial farms due to its efficacy to reduce diarrhea and digestive disorders (Hahn and Baker 1993; Patel et al. 2010; Sargeant et al. 2011; Hu et al. 2013). Pharmacological levels of ZnO improve performance during this period and reduce the effects produced by enterotoxigenic *E. coli* (ETEC), thanks to the inhibition of chloride secretion stimulated by cAMP (Hoque et al. 2005). In addition, ZnO can reduce the bacterial population of the gastrointestinal tract thanks to its bactericidal effects (Söderberg et al. 1990). However, the therapeutic doses of ZnO also have numerous associated problems: toxic effects on the piglet in cases of prolonged administrations, environmental contamination due to the use of manure with high levels of zinc (Poulsen and Larsen 1995; Sargeant et al. 2010), acceleration of the appearance of bacterial resistance to antibiotics and heavy metals (Cavaco et al. 2010, 2011), and changes in the composition of the gastrointestinal bacterial population (Vahjen et al. 2010). For these reasons, the European Union has opted for a ban on its use in high doses from 2022 (Standing Committe on Veterinary Medicinal Products 2017).

Nowadays, some plant extracts are widely used in the livestock sector as feed additives capable of improving the growth performance of animals, representing a possible alternative to ZnO (Allan and Bilkei 2005). Two of these plant extracts are those obtained from oregano (*Origanum vulgare L.)* and garlic (*Allium sativum*).

Carvacrol and thymol are two components that are found in high concentration (78–82%) in oregano (Pandey et al. 2003) and have shown remarkable antimicrobial, antifungal and antioxidant activity in vitro (Daouk et al. 1995; Cervato et al. 2000; Dorman and Deans 2000). Other reports, however, point out that oregano can also cause cell damage (Leyva-López et al. 2017). To preserve the volatile compounds and improve the stability of oregano essential oil (OEO), sunflower oil can be used to micro capsulate it, thus facilitating its handling and administration (Bakry et al. 2015).

Regarding garlic, the degradation products of sulphides such as alliin, diallyl sulphides and allicin (Amagase et al. 2001) form the active compounds, which show beneficial effects thanks to their antimicrobial, antifungal, antiviral, antioxidant and immunomodulation properties (Sallam et al. 2004; Aydın et al. 2005; Amagase 2006; Li et al. 2016).

These plant extracts can influence the intestinal function and histological parameters, such as villi morphometry, presence of goblet cells, intraepithelial lymphocytes, and plasma cells (Hernández-Coronado 2020).

The intestinal villi are covered by enterocytes, cells that actively participate in the absorption of nutrients. Goblet cells located between the enterocytes, are capable of secreting mucus that acts as the first barrier against pathogens (Armocida and Valette 2019). The administration of purple garlic powder (PGP) and OEO in broilers has shown a significant increase in the number of goblet cells (Rojo et al. 2016; Hernández-Coronado 2020).

Intraepithelial lymphocytes are cells found in the epithelium of the villi, and take part in the body's innate immune response. Changes in the number of intraepithelial lymphocytes have been observed both in broilers fed with PGP (Rojo et al. 2016) and in piglets with carvacrol (Michiels et al. 2010).

The intestinal mucosa constitutes the first line of defence against pathogens and commensal microorganisms (Kim et al. 2012b). In the intestinal mucosa, we find a diffuse, non-encapsulated compartment in the lamina propria that includes plasma cells where secretory immunoglobulin A antibodies are produced (Brandtzaeg et al. 2008; Bianco et al. 2014). These cells are mainly found around the intestinal crypts; their function is the production of protective humoral factors that act on the surface of the mucosa (Bianco et al. 2014). The presence of a lower number of IgA-producing cells in the tissues is considered positive, since it indicates a lower inflammatory response in the intestine.

The objective of this study was to evaluate the effect in weaned piglets of feeding different concentrations of OEO and PGP as alternatives to ZnO on intestinal health parameters (goblet cells, intraepithelial lymphocytes, and IgA-producing cells) and growth performance under commercial conditions during the transition.

## Material and methods

Data on the composition of the control diet, the bioactive components of OEO, and the chemical and amino acid composition of PGP are described in Rivera-Gomis et al. (2020).

### Additives and feed composition

The piglets received a commercial base diet formulated to cover all their energy and nutritional needs. From weaning, for two weeks, the piglets received a pre-initiation feed as a base diet. For the next five weeks they were given a starter food, until they were ten weeks old. These diets did not contain any type of antibiotics.

The OEO was purchased from the company Esencias Martínez Lozano S.A (Murcia, Spain), and was administered to the groups in different concentrations: 0.4% and 1.2%. The 10% OEO was encapsulated by a coating of mono and diglycerides of edible fatty acids and hydrogenated sunflower fat in a size of 800 µm. The company in charge of the encapsulation was AT CAPSELOS SL (Huesca, Spain).

The PGP was obtained from Las Pedroñeras "Allibia Fresh Flour" and contained 63% purple garlic in the form of puree and dry powder, with silicic acid (E-551) and citric acid as additives. The garlic was provided by the company Adibio S.L. (Teruel, Spain) and was administered in different concentrations: 0.4% and 2%.

### Animals, housing and experimental design

The University of Murcia, through the Ethical Committee for Animal Experimentation (CEEA), approved the experimental protocols used in the study (Authorization Code 471/2018). Animal handling was carried out in accordance with current legislation on animal welfare in the EU (European Commission 1998, 2008).

The animal population studied was located in the facilities of the company Dalland Hybrid España S.A (DHSA, Murcia, Spain). The piglets were crosses of Pietrain, Large White and Landrace. Weaning was carried out at twenty-one days of age, and slaughter at ten weeks of age. The animals were housed in commercial farm conditions.

There were seven groups according to the additive and dose received: control group (basal diet), ZnO, OEO 0.4% and 1.2%, PGP 0.4% and 2%, and OEO 1.2% + PGP 2%.

ZnO was administered as Zincotrax (Andrés Pintaluba S.A., Tarragona, Spain) at a concentration of 1000 mg of ZnO/g. The final dose was 3100 mg of ZnO/Kg of feed (2500 mg of Zn/Kg of feed). The animals in the ZnO group were the only ones that received zinc in the diet and it was administered during the first two weeks of the transition to later change to the basal diet. On the other hand, the doses of OEO and PGP used in each treatment are similar to those proposed by Rivera-Gomis et al. (2020).

A total of 3000 animals were used through ten replicates. Each replica had the seven treatments. The number of animals used in each treatment and per pen is specified in Table 1. Each replicate lasted seven weeks, from weaning to the end of the transition.

**Table 1:** Experimental design

Treatment	Animals per pen	Animals per replica	Total
Control group	25	50	500
ZnO	25	50	500
OEO 0.4%	15	30	300
OEO 1.2%	25	50	500
PGP 0.4%	15	30	300
PGP 2%	25	50	500
OEO 1.2% + PGP 2%	20	40	400
Total			3000

The initial body weight (BW) of the piglets was 5.70 ± 0.85 kg. The groups had ad libitum access to both food and water through nipple drinkers and feeders. After the end of the research, the animals were sent to a fattening farm to continue their productive cycle under commercial conditions.

### Sample collection and preparation

For the study of the histological parameters, samples of the jejunum and ileum were used. The samples were taken at the end of the experimental period when the piglets were ten weeks old. The total number of animals sacrificed per treatment and replica to obtain the intestinal samples was two. Samples from a total of 20 animals were obtained from each treatment. Therefore, the total number of animals where the histological parameters were studied was 140: 140 jejunum and 140 ileum samples. All the animals sacrificed to obtain samples weighed 20 ± 1 kg.

5 cm of the jejunum were taken at 100 cm from the ileocecal valve. Similarly, 5 cm of the ileum were sectioned at 10 cm from the ileocecal valve. On the sample obtained, a longitudinal cut was made in the middle of the sample, to obtain a closed tubular portion of 2.5 cm and an open portion of the same size. The tissue obtained was placed in a 10% aqueous solution of formaldehyde and sent to the Department of Comparative Anatomy and Pathology, Veterinary Medicine Faculty in Murcia University, where they were kept at room temperature for 48 h.

The samples were cut transversely into segments and placed in plastic cassettes to be reintroduced in 10% formalin. The tissues were then soaked in paraffin for 12 h and allowed to cool for solidification. From the paraffin block, several cuts were made with the microtome and 4 μm samples were obtained, which were placed on slides in two sections (one open and one closed).

Samples for goblet cell and intraepithelial lymphocyte counts were stained with PAS (Periodic Acid Schiff) stain. For immuno-histochemical analysis, the detection of IgA-producing cells was performed by avidin–biotin-peroxidase complex technique, according to de Groot et al. (2021): The samples were deparaffinised and dehydrated with gradual ethanol and the endogenous peroxidase activity was quenched in 3% H2O2 in methanol for 30 min. For antigens recovery, samples were pre-treated with 10% pronase in Tris Buffered Saline (TBS) (Sigma-Aldrich) for 12 min. The samples were then rinsed in TBS (3 × 5 min) and incubated with blocking solution per slide for 30 min at 20 °C in a humid chamber. Subsequently, the samples were incubated for 1 h at 37 °C with the primary antibody (goat anti-pig IgA, Bethyl) diluted 1:2000 in TBS. The secondary anti-body (biotin-conjugated rabbit anti-goat, Dako), diluted 1:250 in TBS, was incubated 30 min at 20 °C. As a vector, the Vectastain Elite ABC Kit was applied for 1 h. at 20ºC. To detect the positive label, 3,3'-diaminobenzidine tetra hydrochloride (Dako) was obtained. Finally, the sections were stitched with Mayer's haematoxylin, dehydrated, and mounted.

The relationship between the type of nutraceutical administered and the population used in the histological study can be seen below in Table 3 and 4.

For the study of the growth performance, a total of 3000 animals were used. The follow-up was carried out until 10 weeks of life. The parameters studied were Average Daily Gain (ADG), Average Daily Feed Intake (ADFI), Feed Conversion Ratio (FCR) and Final Body Weight (FBW).

### Cell count

For the count of goblet cells and intraepithelial lymphocytes, the histological preparations were sent to the Scientific and Technical Research Area (ACTI) of the Murcia University where images were taken and scanned. The images were managed with the Digital Image Hub (DIH) package and the count was made with the Qu Path application (0.2.3).

Goblet cells and intraepithelial lymphocytes present in 10 epithelia of the villi of each scanned sample were counted. The methodology consisted of placing the display at 80 × magnification, taking an image of the epithelium of the villi in the area closest to the base, and rotating the image until it was horizontal.

Immunocytochemistry (IHC) was performed to count IgA-producing cells. The IHC staining technique makes it possible to show the IgA present in the cells of interest using labels. Regarding the study of PAS staining, the images were obtained from the ACTI service of the University of Murcia and the Qu Path application (0.2.3) for counting IgA-producing cells. The count was performed at 80 × magnification, quantifying IgA positive cells present in 10 fields of the lamina propria of the jejunum and ileum.

### Statistical analysis

The statistical variables of intestinal health studied were goblet cells, intraepithelial lymphocytes and IgA-producing cells. The statistical variables of growth performance were ADG, FCR and FBW. All variables were studied from 10 replicates.

The data obtained from the count were analysed using the statistical software IBM SPSS Statistics (version 26.0). Data followed a normal distribution and were presented as mean ± standard error of the mean (SEM) and compared using the one-way ANOVA test, followed by Tukey's multiple comparisons test. The value of p < 0.05 was used to indicate significance in all analyses.

## Results

### Growth performance

Significant differences were found between the experimental groups in relation to the measured growth performance: ADG (*p* < 0.005), FCR (*p* < 0.001) and FBW (*p* < 0.005). No significant differences were found between the average daily feed intake of the different treatments (*p* = 0.063).

In relation to the ADG (SEM = 0.01) (Table 2), the highest values were found in the group that received the OEO 1.2% (0.28), which showed significant differences with the group treated with OEO 0.4% (0.24) and PGP 0.4% (0.23).

**Table 2:** Results of the statistical analysis (the one-way ANOVA test) with the SPSS program (Version 26.0) of the growth performance and consumption: ADG, FCR, ADFI and FBW

Group	R	S/R	S	ADG	FCR	ADFI	FBW
				Mean	Mean	Mean	Mean
Control group	10	50	500	0.27^abc^	2.20^bc^	0.58	18.68^ab^
ZnO	10	50	500	0.28^bc^	1.77^ab^	0.49	19.14^ab^
OEO 0.4%	10	30	300	0.24^ab^	2.16^abc^	0.51	17.35^ab^
OEO 1.2%	10	50	500	0.29^c^	1.84^ab^	0.53	19.76^b^
PGP 0.4%	10	30	300	0.23^a^	2.58^c^	0.59	16.82^a^
PGP 2%	10	50	500	0.28^bc^	1.91^ab^	0.54	19.32^b^
OEO 1.2% + PGP 2%	10	40	400	0.28^bc^	1.68^a^	0.47	19.79^b^
SEM				0.01	0.05	0.01	0.24
P-value				< 0.005	< 0.001	0.063	< 0.005

The letters (a, b, c) indicate statistically significant differences between the groups (*p* ≤ 0.05); *R* Replicate; *S/R* Sample/Replicate; *S* Sample; *ADG* Average Daily Gain; *FCR* Feed Conversion Rate; *FBW* Final Body Weight; *SEM* Standard Error of the Mean

For the FCR (SEM = 0.05) (Table 2), the group that obtained the lowest value was the OEO 1.2% + PGP 2% (1.68), which showed significant differences with Control Group (2.20) and PGP 0.4% (2.58). The ZnO (1.78), OEO 1.2% (1.84) and PGP 2% (1.91) groups also showed significant differences with PGP 0.4%.

Regarding the FBW (SEM = 0.24) (Table 2), the groups that obtained the lowest values were OEO 1.2% + PGP 2% (19.79), OEO 1.2% (19.76) and PGP 2% (19.32). These groups just showed significant differences with the PGP 0.4% (16.82).

### Goblet cells and intraepithelial lymphocytes

Regarding the presence of goblet cells in the epithelium of the villi of the jejunum and ileum (Table 3), statistically significant differences were found between the groups (*p* < 0.001). In the jejunum (SEM = 0.13), the highest levels of goblet cells were reached by PGP 2% (7.22), being the only group that showed significant differences with all groups with lower doses OEO 0.4% (5.30) and PGP 0.4% (4.56) and the Control Group (5.95). ZnO and OEO 1.2% + PGP 2% showed significant differences with OEO 0.4% and PGP 0.4%. The OEO 1.2% only showed significant differences with the PGP 0.4%.

**Table 3:** Results of the statistical analysis (the one-way ANOVA test) with the SPSS program (Version 26.0) of the parameters measured by PAS staining: Jejunum and ileum globet cells and jejunum and ileum intraepithelial lymphocytes

Group	R	S/R	S	JGC	IGC	JIL	IIL
Control Group	10	2	20	5.95^bc^	7.21^ab^	21.62^ab^	23.07^bc^
ZnO	10	2	20	7.00^ cd^	8.14^b^	18.29^a^	18.97^a^
OEO 0.4%	10	2	20	5,30^ab^	6.80^ab^	24.35^b^	26.52^c^
OEO 1.2%	10	2	20	6.14^bcd^	6.96^ab^	20.60^ab^	22.13^ab^
PGP 0.4%	10	2	20	4,56^a^	6.31^a^	22.29^ab^	23.59^bc^
PGP 2%	10	2	20	7.22^d^	7.91^b^	20.78^ab^	21.60^ab^
OEO 1.2% + PGP 2%	10	2	20	6.84^ cd^	6.86^ab^	22.61^b^	23.59^bc^
SEM				0.13	0.13	0.38	0.39
P-value				< 0.001	< 0.001	< 0.001	< 0.001

The letters (a, b, c, d) indicate statistically significant differences (*p* ≤ 0.05); *R* Replicates; *S/R* Samples/Replicates; *S* Samples; *JGC* Jejunum Globet Cells; *IGC* Ileum Globet Cells; *JIL* Jejunum Intraepihthelial Lymphocytes; *IIL* Ileum Intraepithelial Lymphocytes; *SEM* Standard Error of the Mean

In the ileum (SEM = 0.13) there were fewer differences. PGP 2% (7.91) and ZnO (8.14) were the only groups that presented significant differences with respect to PGP 0.4% (6.30).

In relation to the presence of intraepithelial lymphocytes in the jejunum (*p* = 0.001) (SEM = 0.38) (Table 3), significant differences were also found in the jejunum. ZnO (18.29) was the group with the lowest count and the only one that presented significant differences with OEO 0.4% (24.35) and OEO 1 0.2% + PGP 2% (22.61).

Greater differences were observed for the ileum (*p* < 0.001) (SEM = 0.39) (Table 3). ZnO (18.97) had the lowest count and presented significant differences with Control Group (23.07), OEO 1.2% + PGP 2% (23.59), PGP 0.4% (23.59) and OEO 0.4% (26.52). PGP 2% and OEO 1.2% also showed significant differences with OEO 0.4%.

### IgA-producing plasma cell

Regarding the results of the IgA-producing cell counts, statistically significant differences were found between the experimental groups, both in jejunum and ileum (*p* < 0.001).

The experimental groups that presented the lowest number of IgA-producing cells in the jejunum (SEM = 0.50) (Table 4) were the groups OEO 1.2% (2.25) and OEO 1.2% + PGP 2% (2.56), PGP 2% (4.00) and Control Group (4.42). These groups presented significant differences with ZnO (9.71), OEO 0.4% (10.46) and PGP 0.4% (10.89).

**Table 4:** Results of the statistical analysis (the one-way ANOVA test) with the SPSS program (Version 26.0) of the counts of IgA-producing cells measured by IHC staining

Group	R	S/R	S	J (IgA) PC	I (IgA) PC
Control Group	10	2	20	4.42^a^	6.39^abc^
ZnO	10	2	20	9.71^b^	12.38^d^
OEO 0.4%	10	2	20	10.46^b^	9.52^bcd^
OEO 1.2%	10	2	20	2.25^a^	2.60^a^
PGP 0.4%	10	2	20	10.89^b^	9.86^ cd^
PGP 2%	10	2	20	4.00^a^	4.86^a^
OEO 1.2% + PGP 2%	10	2	20	2.56^a^	5.28^ab^
SEM				0.50	0.47
P-value				< 0.001	< 0.001

The letters (a, b, c, d) indicate statistically significant differences (*p* ≤ 0.05); J (IgA) *PC* JejUnum IgA Producing Cells; I (IgA) *PC* Ileum IgA Producing Cells; *SEM* Standard Error of the Mean

More differences were found in the ileum (SEM = 0.47) (Table 4). The groups with the lowest values were the same as in the jejunum. However, OEO 1.2% (2.60) and PGP 2% (4.86) showed significant differences with OEO 0.4% (9.52), PGP 0.4% (9.86) and ZnO. The group OEO 1.2% + PGP 2% (5.28) also showed significant differences with PGP 0.4% and ZnO, and the Control Group (6.39) with the ZnO.

## Discussion

The prohibition of high doses of ZnO in the pig diet is a great health and production challenge (López et al. 2021). Changes in the management and feeding of animals must be addressed correctly. To date, no product studied can be categorized as a real substitute for ZnO. OEO and PGP have been approached as nutritional alternatives to ZnO in order to optimize the feeding of piglets during post-weaning.

In the productive sector, it is necessary that an alternative that helps the physiological maintenance of intestinal functionality does not have a negative impact on growth. In the present research, OEO 1.2%, PGP 2% and their combination showed levels equivalent to or better than ZnO in terms of ADG, FCR and FBW. In poultry production, garlic has been described as an alternative capable of improving growth performance (Miralles et al. 2014; Agulló et al. 2016). Broilers fed different compounds containing garlic have shown increased FBW (Rojo et al. 2016; Giannenas et al. 2019) and better FCR (Giannenas et al. 2019). OEO has also been described as a product capable of improving growth performance in broilers (Tzora et al. 2016; Hernández-Coronado 2020).

Enhancing effects on FBW of garlic supplemented feed have been described in pigs (Tatara et al. 2008; Wang et al. 2011; Liu et al. 2014). Rivera-Gomis et al. (2020) points out an improvement in growth performance (ADG and FBW) in piglets fed low doses of OEO (0.4%) and PGP (0.4%). In fact, in our research, the PGP 0.4% presented the worst results in terms of growth performance. This fact is in agreement with the results obtained in terms of health parameters for the same treatment. Our results are more robust than Rivera-Gomis et al. (2020), since they have been carried out under commercial conditions, with a large pig population and a greater number of animals per pen. However, this situation highlights the need to continue investigating the effects of these additives in order to establish the most appropriate doses.

In short, OEO 1.2%, PGP 2% and their combination, used in post-weaning piglet feeding, show favourable results in accordance with those published in other studies on broilers. However, these compounds have not been extensively studied in pigs. This fact, together with a predominance of studies under experimental and non-commercial conditions, highlights the importance of this research.

In agreement with the productive results, the intestinal health parameters measured showed that the doses of OEO 1.2% and PGP 2%, as well as their combination, exerted a similar effect to ZnO in the jejunum and ileum, promoting the production of goblet cells. It should be noted that in the ileum, OEO 0.4% also showed favourable results, which could reflect the possibility of adjusting the OEO dose more efficiently.

In relation to PGP, our results are in line with those of Giannenas et al. (2019) who pointed out a significant increase in the number of goblet cells in broilers fed with an herbal compound, among whose components was *Allium sativum*, and by Rojo et al. (2016) who determined a significantly higher count of goblet cells in broilers fed 2% garlic. In addition, other author such as Zhao et al. (2010) also reported an increase in mucin expression (secreted by goblet cells) in the epithelial barrier in his respective studies with rats.

Regarding OEO, our results do not differ from those of Liu et al. (2019) and Tzora et al. (2016) who indicated an increase in the number of goblet cells in broilers that received oregano through their diet. Hernández-Coronado (2020) also described a significant increase in goblet cells in broilers that received OEO through drinking water alone and drinking water and diet combined.

With respect to the presence of intraepithelial lymphocytes in the jejunum and ileum, the presence of lymphocytes at high levels is considered negative for the animal, as it reflects the presence of inflammation in the mucosa (Hayday et al. 2001), and a higher rate of enterocyte renewal (Guy-Grand et al. 1998). This may indicate a higher level of stress in the animal and a reduction in digestive efficiency and absorption of nutrients, which can have a negative impact on growth and productivity (Michiels et al. 2010).

The results obtained for the intraepithelial lymphocyte count are in line with those obtained for the count of goblet cells. The lowest values were found in the animals that received ZnO. In the jejunum, the OEO 1.2% showed results closer to ZnO, although these were very similar to those of the PGP 2%. No significant differences were found between the ZnO and the high doses of PGP and OEO, the PGP 0.4% and the Control Group.

In the ileum, however, only the high doses of PGP and OEO did not show significant differences with ZnO. Again, the results could indicate that high doses of additives exert a beneficial effect on intraepithelial lymphocyte production in the jejunum and ileum similar to that of ZnO. Michiels et al. (2010), as in our study, reported a decrease in the number of intraepithelial lymphocytes in weaned piglets fed 500 mg/Kg and 2000 mg/Kg of carvacrol.

Regarding IgA-producing cells, the presence of a lower number can be considered positive, since it indicates a lower inflammatory response in the intestine, and therefore, a better intestinal function that is not reduced by the action of counterproductive processes such as inflammation of the intestinal tissues (Liu et al. 2019; Van Nevel et al. 2003).

In our study, ZnO showed intermediate levels of IgA-producing cells compared to the additive groups, so our main interest was in obtaining results close to those of ZnO. In the jejunum, the high doses of PGP and OEO, their combination and the Control Group showed the lowest counts.

In the ileum, all groups had results lower than those of ZnO. The order was similar to the jejunum and the low doses of PGP and OEO showed results more similar to ZnO, without significant differences. The high doses of PGP and OEO, their combination. The high doses of PGP, OEO and their combination showed, again, the best results.

The results could be variably interpreted. Although low doses of OEO and PGP obtained results more similar to those of ZnO, high doses of the studied additives and their combination lower the inflammatory response in both the jejunum and ileum by reducing the need for IgA antibody production by plasma cells. This would be a positive effect of high doses of PGP and OEO, since the production of IgA in the intestine is reduced, which is indicative of a reduction in physiological stress in piglets, which has a positive impact on growth performance (de Groot et al. 2021).

Finally, it is important to point out that although the combination of PGP and OEO showed favorable results, no synergistic action was observed. This could be due to the fact that excess supplementation of these compounds can lead to inflammatory problems that reduce their beneficial potential (Vega Montalvo and Carrillo Domínguez 1997; Rivera-Gomis et al. 2020).

## Conclusion

OEO 1.2%, PGP 2% and their combination are capable of improving the structural and immunological integrity of the intestinal mucosa under commercial conditions without negatively affecting growth performance. In high doses, these additives have an anti-inflammatory effect in the small intestine and improve the state of the intestinal epithelium, increasing the production of goblet cells and reducing the number of intraepithelial lymphocytes. In addition, they are capable of reducing IgA production in the jejunum and ileum, reducing inflammation and physiological stress in piglets, achieving a growth performance similar to that of piglets treated with ZnO during transition.

## Data Availability

The datasets used and analysed during the current study are available from the corresponding author on reasonable request.

## Abbreviations

OEO: Oregano Essential Oil
PGP: Purple Garlic Powder
PAS: Periodic Acid Schiff
TBS: Tris Buffered Saline
ADG: Average Daily Gain
FCR: Feed Conversion Ratio
FBW: Final Body Weight
DIH: Digital Image Hub
IHC: Immunocytochemistry

## References

[CR1] Agulló V, Miralles A, Bernabé A, Cubero MJ (2016). Efectos sanitarios y productivos del ajo morado en avicultura industrial y ecológica. An Vet Mur.

[CR2] Allan P, Bilkei G (2005). Oregano improves reproductive performance of sows. Theriogenology.

[CR3] Amagase H (2006). Clarifying the real bioactive constituents of garlic. J Nutr.

[CR4] Amagase H, Petesch BL, Matsuura H, Kasuga S, Itakura Y (2001). Intake of garlic and its bioactive components. J Nutr.

[CR5] Armocida A, Valette E (2019) Salud intestinal en el cerdo. Vetanco. https://www.vetanco.com/es/wp-content/uploads/sites/3/2019/04/A.C.-SALUD-INSTESTINAL-DEL-CERDO.pdf. Accesed 16 Jan 2021

[CR6] Aydın S, Başaran AA, Başaran N, Aydin S (2005). Modulating effects of thyme and its major ingredients on oxidative DNA damage in human lymphocytes. J Agric Food Chem.

[CR7] Bakry AM, Abbas S, Ali B, Majeed H, Abouelwafa MY, Mousa A, Liang L (2015). Microencapsulation of oils: A comprehensive review of benefits, techniques, and applications. Compr Rev Food Sci Food Saf.

[CR8] Bianco C, Felice V, Panarese S, Marrocco R, Ostanello F, Brunetti B, Muscatello LV, Leotti G, Vila T, Joisel F (2014). Quantitative immunohistochemical assessment of IgA, IgM, IgG and antigen-specific immunoglobulin secreting plasma cells in pig small intestinal lamina propria. Vet Immunol Immunopathol.

[CR9] Brandtzaeg P, Kiyono H, Pabst R, Russell MW (2008). Terminology: nomenclature of mucosa-associated lymphoid tissue. Mucosal Immunol.

[CR10] Cavaco LM, Hasman H, Stegger M, Andersen PS, Skov R, Fluit AC, Ito T, Aarestrup FM (2010). Cloning and occurrence of czrC, a gene conferring cadmium and zinc resistance in methicillin-resistant *Staphylococcus aureus* CC398 isolates. Antimi-Crob Agents Chemother.

[CR11] Cavaco LM, Hasman H, Aarestrup FM (2011). Zinc resistance of *Staphylococcus aureus* of animal origin is strongly associated with methicillin resistance. Veter Microbiol.

[CR12] Cervato G, Carabelli M, Gervasio S, Cittera A, Cazzola R, Cestaro B (2000). Antioxidant properties of oregano (*Origanum vulgare*) leaf extracts. J Food Biochem.

[CR13] Daouk RK, Dagher SM, Sattout EJ (1995). Antifungal activity of the essential oil of *Origanum syriacum* L. J Food Prot.

[CR14] de Groot N, Fariñas F, Cabrera-Gómez CG, Pallares FJ, Ramis G (2021). Weaning causes a prolonged but transient change in immune gene expression in the intestine of piglets. J Anim Sci.

[CR15] Dorman HJD, Deans SG (2000). Antimicrobial agents from plants: antibacterial activity of plant volatile oils. J Appl Microbiol.

[CR16] European Commission (1998) Council Directive 98/58/EC of 20 July 1998 concerning the protection of animals kept for farming purposes. Off J L 221:23–27

[CR17] European Commission (2008) Council Directive 2008/120/EC of 18 December 2008 laying down minimum standards for the protection of pigs. Off J L 47:5–13

[CR18] Giannenas I, Bonos E, Filliousis G, Stylianaki I, Kumar P, Lazari D, Christaki E, Florou-Paneri P (2019). Effect of a polyherbal or an arsenic-containing feed additive on growth performance of broiler chickens, intestinal microbiota, intestinal morphology, and lipid oxidation of breast and thigh meat. J Appl Poult.

[CR19] Guy-Grand D, DiSanto JP, Henchoz P, Malassis-Séris M, Vassalli P (1998). Small bowel enteropathy: role of intraepithelial lymphocytes and of cytokines (IL-12, IFN-γ, TNF) in the induction of epithelial cell death and renewal. Eur J Immunol.

[CR20] Hahn JD, Baker DH (1993). Growth and plasma zinc responses of young pigs fed pharmacologic levels of zinc. J Anim Sci.

[CR21] Hayday A, Theodoridis E, Ramsburg E, Shires J (2001). Intraepithelial lymphocytes: exploring the Third Way in immunology. Nat Immunol.

[CR22] Hernández-Coronado AC (2020) Evaluación del aceite de orégano (*Lippia berlandieri Schauer*) sobre el comportamiento productivo de pollos de engorde. In: Repositorio Académico Digital. Universidad Autónoma de Nuevo León. http://eprints.uanl.mx/19982/1/1080314465.pdf. Accesed 16 Jan 2021

[CR23] Hoque KM, Rajendran VM, Binder HJ (2005). Zinc inhibits cAMP-stimulated Cl secretion via basolateral K-channel blockade in rat ileum. Am J Physiol.

[CR24] Hu C, Song J, Li Y, Luan Z, Zhu K (2013). Diosmectite–zinc oxide composite improves intestinal barrier function, modulates expression of pro-inflammatory cytokines and tight junction protein in early weaned pigs. Br J Nutr.

[CR25] Kim J, Hansen C, Mullan B, Pluske J (2012). Nutrition and pathology of weaner pigs: Nutritional strategies to support barrier function in the gastrointestinal tract. Anim Feed Sci Technol.

[CR26] Kim SH, Lee KY, Jang YS (2012). Mucosal immune system and M cell-targeting strategies for oral mucosal vaccination. Immune Netw.

[CR27] Leyva-López N, Gutiérrez-Grijalva EP, Vazquez-Olivo G, Heredia JB (2017). Essential oils of oregano: biological activity beyond their antimicrobial properties. Molecules.

[CR28] Li WR, Shi QS, Dai HQ, Liang Q, Xie XB, Huang XM, Zhao GZ, Zhang L (2016). Antifungal activity, kinetics, and molecular mechanism of action of garlic oil against *Candida albicans*. Sci Rep.

[CR29] Liu Y, Song M, Che TM, Lee JJ, Bravo D, Maddox CW, Pettigrew JE (2014). Dietary plant extracts modulate gene expression profiles in ileal mucosa of weaned pigs after an Escherichia coli infection. J Anim Sci.

[CR30] Liu SD, Song MH, Yun W, Lee JH, Lee CH, Kwak WG, Oh HJ, Kim HB, Cho JH (2019). Effects of oral administration of various essential oils on blood metabolites, intestine development, microbial enumeration, and meat quality in broilers. Indian J Anim Res.

[CR31] López M, Madrid J, Hernández F, Ros MA, Segura JC, López MJ, Pallarés FJ, Sánchez CJ, Martínez-Miró S (2021). Effect of feed supplementation with *Clostridium butyricum*, alone or in combination with carob meal or citrus pulp, on digestive and metabolic status of piglets. Animals.

[CR32] Michiels J, Missotten J, Van Hoorick A, Ovyn A, Fremaut D, De Smet S, Dierick N (2010). Effects of dose and formulation of carvacrol and thymol on bacteria and some functional traits of the gut in piglets after weaning. Arch Anim Nutr.

[CR33] Miralles A, Otal J, Palacios C, Martín P, León L, Cubero MJ (2014) Evaluación *in vivo* de la actividad antibacteriana y promotora del crecimiento de la moltura de ajo (*ZooAllium*) en pollos ecológicos. Exponed at the XIX Simposio Anual de Asociación de Veterinarios Especialistas en diagnóstico laboratorial (AVEDILA), Zaragoza, Spain

[CR34] Moberg GP, Mench JA (2000) The biology of animal stress: basic principles and implications for animal welfare. Wallingford, Oxfordshire. 10.1079/9780851993591.0000

[CR35] Pandey A, Rai M, Acharya D (2003). Chemical composition and antimycotic activity of the essential oils of corn mint (*Mentha arvensis*) and lemon grass (*Cymbopogon flexuosus*) against human pathogenic. Fungi Pharm Biol.

[CR36] Patel A, Mamtani M, Dibley MJ, Badhoniya N, Kulkarni H (2010). Therapeutic value of zinc supplementation in acute and persistent diarrhea: a systematic review. PLoS ONE.

[CR37] Pié S, Lallès JP, Blazy F, Laffitte J, Sève B, Oswald IP (2004). Weaning is associated with an upregulation of expression of inflammatory cytokines in the intestine of piglets. J Nutr.

[CR38] Poulsen HD, Larsen T (1995). Zinc excretion and retention in growing pigs fed increasing levels of zinc oxide. Livest Prod Sci.

[CR39] Rivera-Gomis J, Peres C, Martínez-Conesa C, Otal J, Cerón JJ, Escribano D, Cubero MJ (2020). Effects of dietary supple-mentation of garlic and oregano essential oil on biomarkers of oxidative status, stress and inflammation in post-weaning piglets. J Anim Sci.

[CR40] Rojo E, Miralles A, Bernabé A, Cubero MJ (2016). Efecto del ajo morado en el rendimiento productivo y en la salud intestinal de pollos de engorde en producción industrial y ecológica. An Vet Mur.

[CR41] Sallam KH, Ishioroshi IM, Samejima K (2004). Antioxid antimicrob Eff garlic chick sausage. Leb Wiss U Technol.

[CR42] Sargeant HR, McDowall KJ, Miller HM, Shaw MA (2010). Dietary zinc oxide affects the expression of genes associated with inflammation: transcriptome analysis in piglets challenged with ETEC K88. Veter Immunol Immunopathol.

[CR43] Sargeant HR, Miller HM, Shaw MA (2011). Inflammatory response of porcine epithelial IPEC J2 cells to enterotoxigenic *E. coli* infection is modulated by zinc supplementation. Mol Immunol.

[CR44] Söderberg TA, Sunzel B, Holm S, Elmros T, Hallmans G, Sjöberg S (1990). Antibacterial effect of zinc oxide in vitro. Scand J Plast Reconstr Surg Hand Surg.

[CR45] Standing Committee on Veterinary Medicinal Products (2017) https://ec.europa.eu/health/documents/community-register/2017/20170626136754/dec_136754_en.pdf. Accessed 11 Nov 2021

[CR46] Tatara MR, Sliwa E, Dudek K, Gawron A, Piersiak T, Dobrowolski P, Mosiewicz J, Siwicki A, Studzinski T (2008). Aged garlic extract and allicin improve performance and gastrointestinal tract development of piglets reared in artificial sow. Ann Agric Environ Med.

[CR47] Tzora A, Giannenas I, Karamoutsios A, Papaioannou N, Papanastasiou D, Bonos E, Skoufos S, Bartzanas T, Skoufos I (2016). Effects of oregano, attapulgite, benzoic acid and their blend on chicken performance, intestinal microbiology, and intestinal morphology. J Poult Sci.

[CR48] Vahjen W, Pieper R, Zentek J (2010). Bar-coded pyrosequencing of 16S rRNA gene amplicons reveals changes in ileal porcine bacterial communities due to high dietary zinc intake. Appl Environ Microbiol.

[CR49] Van Nevel CJ, Decuypere JA, Dierick N, Molly K (2003). The influence of *Lentinus edodes* (Shiitake mushroom) preparations on bacteriological and morphological aspects of the small intestine in piglets. Arch Tierernahr.

[CR50] Vega Montalvo R, Carrillo Domínguez C (1997). Efecto sobre la motilidad intestinal y toxicidad aguda oral del extracto fluido de Ocimum gratissimum L. (orégano cimarrón). Rev Cuba Plantas Med.

[CR51] Wang JP, Yoo JS, Jang HD, Lee JH, Cho JH, Kim IH (2011). Effect of dietary fermented garlic by Weissella koreensis powder on growth performance, blood characteristics, and immune response of growing pigs challenged with *Escherichia coli* lipopoly-saccharide. J Anim Sci.

[CR52] Wijtten PJA, Van Der Meulen J, Verstegen MWA (2011). Intestinal barrier function and absorption in pigs after weaning: A review. Br J Nutr.

[CR53] Zhao TP, Zhou S, Qin SD (2010). The regulating effect on different moxibustion for colonic mucosa mucin in rats with ulcerative colitis. Chinese Arc Tradit Chinese Med.

[CR54] Zhu H, Jia Z, Misra H, Li YR (2012). Oxidative stress and redox signalling mechanisms of alcoholic liver disease: Updated experimental and clinical evidence. J Dig Dis.

